# Silymarin attenuates diabetic nephropathy in rats via modulation of the miRNA-223/NLRP3/caspase-1/GSDMD axis and inflammasome-related pyroptotic signaling

**DOI:** 10.1186/s13062-026-00857-6

**Published:** 2026-06-24

**Authors:** Merna G. Aboismaiel, Mohamed N. Amin, Laila A. Eissa

**Affiliations:** Biochemistry Department, Faculty of Pharmacy, Mansoura University, Mansoura, 35516 Egypt

**Keywords:** Diabetic nephropathy, miRNA-223, NLRP3 inflammasome, GSDMD, Silymarin, Pyroptosis

## Abstract

Diabetic nephropathy (DN) is a progressive microvascular complication of diabetes and a leading cause of end-stage renal disease, with limited disease-modifying therapeutic options. DN is characterized by progressive renal dysfunction, oxidative stress, inflammation, fibrosis, and activation of inflammasome-related pathways. Silymarin, a natural compound with antioxidant and anti-inflammatory properties, has demonstrated renoprotective potential; however, its association with miRNA-223/NLRP3/caspase-1/GSDMD signaling in DN remains incompletely defined. Therefore, the present study investigated the potential renoprotective effects of silymarin in a high-fat diet/streptozotocin (HFD/STZ)-induced rat model of DN, with particular focus on miRNA-223 expression and inflammasome-associated pyroptotic signaling. Diabetic rats received oral silymarin (100 mg/kg/day) either for 12 weeks as an early intervention or 4 weeks as a delayed treatment. Renal function indices, blood glucose, HbA1c, serum insulin, HOMA-IR, LDH, lipid profile, body weight, kidney weight/body weight index, oxidative stress markers, and histopathological changes were evaluated. Renal expression of miRNA-223, NOD-like receptor family pyrin domain-containing 3 (NLRP3), caspase-1, gasdermin D (GSDMD), nuclear factor-kappa B (NF-κB) (p65), hypoxia-inducible factor-1 alpha (HIF-1α), tumor necrosis factor-alpha (TNF-α), interleukin-1 beta (IL-1β), and fibronectin was assessed using RT-qPCR, ELISA, and immunohistochemistry. Silymarin significantly improved renal function, glycemic control, insulin resistance, lipid profile, oxidative stress, inflammatory mediators, HIF-1α expression, LDH activity, histopathological injury, and fibrosis relative to untreated DN rats. Moreover, silymarin was associated with increased renal miRNA-223 expression and reduced expression of NLRP3, caspase-1, and GSDMD. These effects were more pronounced in the early intervention group compared with delayed treatment. In conclusion, silymarin attenuated experimental DN via modulation of the miRNA-223/NLRP3/caspase-1/GSDMD axis and suppression of pyroptosis-associated signaling, alongside improvement of oxidative stress, inflammation, hypoxia, fibrosis, and metabolic disturbances. Further mechanistic studies are warranted to clarify the causal relationship between miRNA-223 modulation and the observed renoprotective effects of silymarin.

## Graphical abstract

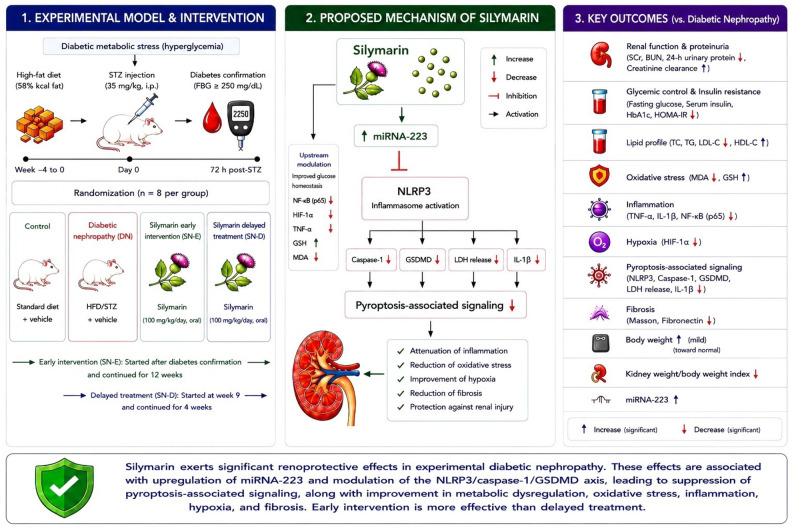

## Introduction

Diabetic nephropathy (DN) is a major microvascular complication of diabetes mellitus and a leading cause of end-stage renal disease (ESRD). The typical course of DN initially involves glomerular hyperfiltration and the onset of albuminuria, which are subsequently followed by a gradual deterioration in renal function [1]. The pathogenesis of DN is driven by chronic hyperglycemia-associated metabolic dysfunction, oxidative stress, inflammation, and progressive structural damage to renal tissue [2]. The increasing global prevalence of type 2 diabetes mellitus (T2DM), which accounts for the majority of diabetes cases, has significantly amplified the burden of DN worldwide [3].

Inflammation-induced renal injury is a central mechanism in DN progression, with particular involvement of the NOD-like receptor family pyrin domain-containing 3 (NLRP3) inflammasome-mediated pyroptotic pathway (NLRP3/caspase-1/GSDMD axis) [4]. In this pathway, nuclear factor-kappa B (NF-κB) functions primarily as an upstream priming signal that is activated after pattern recognition receptors (PRRs) receive danger signals. NF-κB activation is associated with its nuclear translocation and induction of transcriptional upregulation of NLRP3 and pro-IL-1β [5]. Subsequent activation of the NLRP3 inflammasome is triggered by cellular stress signals such as mitochondrial reactive oxygen species (ROS) and ionic imbalance. Activated NLRP3 assembles with the adaptor protein apoptosis-associated speck-like protein containing a caspase recruitment domain (ASC), leading to caspase-1 activation, maturation of IL-1β and IL-18, and cleavage of gasdermin D (GSDMD), ultimately resulting in pyroptotic cell death and amplification of renal inflammation [6].

Pyroptosis is a recently recognized form of programmed inflammatory cell death characterized by features of necrosis. It is driven by a sequence of molecular events that include NLRP3 inflammasome activation, pore formation in the plasma membrane mediated by GSDMD, and the release of pro-inflammatory cytokines. Pyroptosis can proceed via either the canonical pathway, involving caspase-1, or the non-canonical pathway mediated by caspases-4, -5, or -11. These caspases cleave GSDMD, generating an N-terminal fragment that oligomerizes and inserts into the cell membrane to form pores. The formation of these transmembrane pores compromises membrane integrity and disrupts osmotic balance, ultimately leading to cell swelling and lysis. Consequently, intracellular contents, including the pro-inflammatory cytokines IL-1β and IL-18, are released, amplifying the inflammatory response [7].

In addition to inflammatory injury, hypoxia represents a fundamental pathological feature in diabetic kidneys, arising from an imbalance between oxygen supply and demand. While renal oxygen delivery is largely dependent on blood flow, oxygen consumption is primarily driven by tubular sodium reabsorption. In the diabetic state, this balance is disrupted, as hyperglycemia-induced microvascular damage compromises oxygen delivery, whereas enhanced sodium reabsorption, driven by sodium-glucose cotransporter (SGLT) upregulation and glomerular hyperfiltration, increases metabolic demand [8].

This hypoxic milieu promotes stabilization and sustained activation of hypoxia-inducible factor-1 alpha (HIF-1α), which contributes to disease progression by modulating inflammatory, metabolic, and fibrotic pathways [9]. Importantly, emerging evidence indicates a bidirectional crosstalk between hypoxia and inflammatory signaling, particularly pathways governing NF-κB and inflammasome activation. This interaction amplifies renal injury and establishes a self-sustaining cycle of hypoxia, inflammation, extracellular matrix accumulation, and fibrotic remodeling, ultimately leading to progressive nephron loss [10, 11].

At the post-transcriptional level, microRNAs (miRNAs) have emerged as critical regulators of gene expression in DN [12]. Among them, miRNA-223 has been identified as a direct negative regulator of NLRP3, where it suppresses inflammasome assembly and downstream caspase-1 activation and GSDMD-mediated pyroptosis. Thus, miRNA-223 represents a key molecular checkpoint controlling inflammatory cell death and renal injury progression [13].

Despite advances in glycemic control strategies, the incidence and progression of DN continue to rise, and current therapies remain limited by incomplete efficacy and potential adverse effects [14]. This highlights the urgent need for novel multi-target therapeutic approaches targeting the underlying molecular drivers of disease progression.

Silymarin, a flavonolignan complex derived from *Silybum marianum*, possesses well-documented antioxidant, anti-inflammatory, and immunomodulatory properties [15]. Silymarin is widely used as a hepatoprotective agent in the management of chronic liver diseases, including cirrhosis, hepatitis, and non-alcoholic fatty liver disease, where it has been shown to improve liver function and histopathological outcomes [16]. Previous studies have demonstrated its renoprotective effects through attenuation of oxidative stress, lipid peroxidation, and fibrotic signaling [17, 18]. However, its role in regulating miRNA-mediated control of inflammasome activation and pyroptotic cell death in DN remains largely unexplored.

Accordingly, this study aimed to evaluate the renoprotective effects of silymarin in early and delayed treatment interventions in a high-fat diet/streptozotocin (HFD/STZ)-induced rat model of DN, with particular focus on its association with modulation of the miRNA-223/NLRP3/caspase-1/GSDMD axis and pyroptosis-associated signaling, as well as related inflammatory, oxidative, hypoxic, and fibrotic alterations.

## Materials and methods

### Drugs and chemicals

STZ (CAS no.: 18883-66-4) and silymarin (CAS no: 65666-07-1) were obtained from Sigma Aldrich (USA). Citric acid, sodium citrate, and carboxymethyl cellulose (CMC) were purchased from El-Gomhouria (Mansoura, Egypt). Phosphate-buffered saline (PBS) was supplied by Biodiagnostic (Giza, Egypt). All chemicals and reagents used were of analytical grade quality.

### Animals

Ethical approval was granted by the Research Ethics Committee of the Faculty of Pharmacy, Mansoura University, Egypt (Approval No. 2024-07). All procedures complied with ARRIVE guidelines and the National Research Council’s Guide for the Care and Use of Laboratory Animals. Adult male Sprague-Dawley rats (180–220 g) were acclimatized for two weeks under controlled laboratory conditions (22 ± 2 °C, 12 h light/dark cycle) with unrestricted access to standard chow and water. Only male rats were used in the present study to minimize potential variability related to hormonal fluctuations and because male rodents are generally more susceptible to HFD/STZ-induced metabolic and renal alterations in experimental DN models, thereby providing a more consistent experimental DN phenotype [19–21].

### Induction of type-2 diabetes mellitus

Type 2 diabetes was induced by combining a high-fat diet (HFD; 58% fat, 25% protein, 17% carbohydrates) for four weeks followed by a single intraperitoneal injection of streptozotocin (35 mg/kg), freshly dissolved in ice-cold citrate buffer (0.1 M, pH 4.5), after overnight fasting [22]. After 72 h, diabetes diagnosis was verified by checking blood glucose levels with a glucometer. Rats were classified as diabetic if their blood glucose levels reached 250 mg/dl or higher [23]. Diabetic animals continued on HFD throughout the experimental period. Early intervention regimen started 3 days after STZ administration and lasted 12 weeks, whereas the delayed treatment regimen began at week 9 and continued for 4 weeks (from the 9th to the 12th week).

### Experimental design

The experiment consisted of a total of thirty-two rats in four groups, eight rats each. Control group: rats were given a typical rat pellet diet. Then a single i.p. dose of citrate buffer (0.1 M, pH 4.5) was injected into rats after 4 weeks. Three days later, they were administered a daily oral gavage of 0.5% CMC for 12 weeks. Diabetic nephropathy (DN) group: untreated diabetic rats received a daily oral gavage of 0.5% CMC for 12 weeks. Silymarin early intervention (SN-E) group: diabetic rats received silymarin (100 mg/kg) [24] daily as an oral gavage in 0.5% CMC for 12 weeks. Silymarin delayed treatment (SN-D) group: diabetic rats received a daily oral gavage of 0.5% CMC for 8 weeks then received silymarin (100 mg/kg) in 0.5% CMC for 4 weeks starting from the 9th week till the 12th week.

### Sample collection

Upon experiment completion, urine samples were collected from each rat for 24 h using metabolic cages (Nalgene, Rochester, NY, USA). Subsequently, the rats were fasted overnight and their weights were recorded. Thiopental anesthesia (40 mg/kg) was administered i.p. to allow the withdrawal of blood from the retro-orbital plexus. Blood samples were divided into EDTA-containing tubes for glycated hemoglobin (HbA1c) determination and plain tubes for serum separation and biochemical analysis. For serum separation, blood samples were centrifuged at 3,000 rpm for 15 min at 4 °C, and serum was stored at -20 °C. Following euthanasia by decapitation, both kidneys were excised and weighed. Kidney weight/body weight ratio was subsequently calculated as an index of renal hypertrophy. The left kidney was divided into two portions: one snap-frozen in liquid nitrogen for biochemical assays and the other preserved at -80 °C for gene expression analysis. The right kidney was fixed in 10% neutral-buffered formalin for histological and immunohistochemical studies.

### Biochemical analysis in blood and serum

Whole blood samples were collected in EDTA-containing tubes for determination of glycated hemoglobin (HbA1c). HbA1c % was measured using a commercially available assay kit (Crystal Chem, USA, Cat. No. 80300) according to the manufacturer’s instructions. Serum levels of fasting glucose, triglycerides, total cholesterol, HDL-cholesterol, creatinine, blood urea nitrogen (BUN) and lactate dehydrogenase (LDH) activity were measured using spectrophotometric methods and commercial kits (Biodiagnostic, Giza, Egypt) according to manufacturer instructions. Serum insulin levels were determined using rat-specific ELISA kit (ThermoFisher Scientific, USA, Cat. No. ERINS). LDL-cholesterol was calculated using the Friedewald formula [25]. Homeostatic Model Assessment of Insulin Resistance (HOMA-IR) was assessed using the following equation:

$$\:\frac{Fasting\:Insulin\:\left({\upmu\:}IU/mL\right)xFasting\:glucose\:\left(mg/dl\right)}{405}$$

### Urine analysis

Urine samples were centrifuged at 2000 rpm for 10 min at 4 °C. The supernatant was used to determine urinary protein and creatinine concentrations using commercial kits (Biodiagnostic, Egypt). Twenty-four-hour urinary protein excretion (mg/24-hr) and creatinine clearance (ml/min) were subsequently calculated [23].

### Kidney homogenate preparation

Renal tissue was rinsed in ice-cold PBS (pH 7.4) to remove blood residues, weighed, and homogenized to obtain a 10% (w/v) suspension. Homogenization was performed in PBS using a glass homogenizer followed by brief sonication (1 min). The homogenate was centrifuged at 10,000 rpm for 5 min at 4 °C, and the resulting supernatant was collected for further analyses [26].

#### Enzyme-linked immunosorbent assay (ELISA)-based measurements

The renal NLRP3, caspase-1, TNF-α, IL-1β, and GSDMD concentrations were measured using commercial ELISA kits obtained from Aviva Systems Biology (Cat. No. OKCD04232-48), Biovision (Cat. No. E4594-100), MyBioSource (Cat. No. MBS2507393), Abcam (Cat. No. ab100768), and MyBioSource (Cat. No. MBS2705517); respectively. All assays were performed as per manufacturer’s guidelines. Total protein concentration in kidney homogenates was determined by the Bradford method (1976) [27] utilizing a Bradford Assay Kit from Abcam (Cat. No. ab102535).

#### Determination of oxidative stress

For the evaluation of lipid peroxidation and oxidative stress, levels of malondialdehyde (MDA) and glutathione (GSH) were determined in renal homogenate using Biodiagnostic spectrophotometric kits, guided by the manufacturer’s instructions.

### Histopathology of renal tissue

Formalin was used for fixation of the right kidney. The fixed kidney was sectioned longitudinally and embedded in paraffin. Two slide sets were prepared; each had a distinct staining of a 5-µm-thick piece of renal tissue. Histopathological alterations were assessed semi-quantitatively using hematoxylin and eosin (H&E) stain and scores were given from 0 to 3, where 0 is normal, 1 is mild, 2 is moderate, and 3 is severe [28]. Masson’s trichrome staining was utilized for renal fibrosis assessment [29]. A digital camera from Nikon was utilized along with computer software to capture images. The extent of Masson’s-positive area was assessed via Image J analysis software (NIH, USA). The histologist was kept unaware of the experimental groups and a random examination of the slides was carried on.

### Immunohistochemical assays

Renal tissue sections were used for immunohistochemical analysis of NF-κB (p65) and fibronectin. Sections were deparaffinized, rehydrated in graded ethanol, and treated with H_2_O_2_/methanol to block endogenous peroxidase activity. Slides were incubated overnight at 4 °C with primary antibodies against NF-κB (p65) and fibronectin (1:100 dilution) obtained from Santa Cruz Biotechnology, USA (Cat. No. sc-8008 and Cat. No. sc-8422), respectively. Following a PBS rinse, slides were incubated for thirty minutes with an IgG secondary antibody (Abcam, USA; Cat. No. ab97023). Visualization was done via diaminobenzidine, and Mayer’s hematoxylin was utilized for counterstaining. Negative controls were prepared using normal rat serum instead of the primary antibody. Immunostaining showed distinctive brown reactions of both NF-κB (p65) and fibronectin. Quantification of positive staining was carried out using ImageJ (NIH, USA) [30, 31].

### Quantitative reverse-transcription polymerase chain reaction (RTq-PCR)

Total RNA was extracted from the renal tissue using the miRNeasy Mini Kit from Qiagen (Catalog No. 217004). cDNA synthesis from mRNA and miRNA was performed using the QuantiTect Reverse Transcription Kit (Catalog No. 205311) and miRCURY LNA RT Kit (Catalog No. 339340), respectively. The PikoRealTM Real-time PCR System from Thermo Fisher Scientific was used for the detection of mRNA and miRNA using the QuantiTect SYBR Green RT-PCR Kit (Catalog No. 204243) and miRCURY LNA SYBR Green PCR Kit (Catalog No. 339345), respectively. GAPDH and U6 snRNA served as internal controls for mRNA and miRNA normalization. Specific primers for NF-κB (p65), NLRP3, caspase-1, HIF-1α, and GAPDH genes **(**Table 1**)** were designed based on gene sequences from the GenBank and analyzed using NetPrimer (PREMIER Biosoft, USA). Qiagen’s miRCURY LNA miRNA PCR assays were used to analyze the expression of miRNA. The following primer sets were used: rno-miR-223-3p, and U6 snRNA. Relative expression was calculated using the 2^−ΔΔCT^ method.

**Table 1:** Primer sequences of specific genes

Gene of Interest		Primer Sequence	Reference Sequence
NF-κB (p65)	Forward	5`- TGTGTGAAGAAGCGAGACCTG − 3`	NM_199267.2
	Reverse	5`- AAAATCGGATGCGAGAGGAC − 3`	
NLRP3	Forward	5`- GTAGGTGTGGAAGCAGGACT − 3`	NM_001191642.1
	Reverse	5`- CCTTTGCTCCAGACCCTACA − 3`	
Caspase-1	Forward	5`- CGTCTTGCCCTCATTATCTGC − 3`	NM_012762.3
	Reverse	5`- ACAGTATACCCCAGATCCTGC − 3`	
HIF-1α	Forward	5`- GCATCTCCACCTTCTACCCA − 3`	NM_024359.2
	Reverse	5`- TCTGTCTGGTGAGGTTGTCC − 3`	
GAPDH	Forward	5`- CCATCAACGACCCCTTCATT − 3`	NM_017008.4
	Reverse	5`- CACGACATACTCAGCACCAGC − 3`	

The sequences of the primers are designed according to the GenBank and the reference sequences of the genes of interest are mentioned. NF-κB (p65): Nuclear factor-kappa B (p65), NLRP3: NOD-like receptor family pyrin domain-containing 3, HIF-1α: Hypoxia-inducible factor-1 alpha, GAPDH: Glyceraldehyde-3-phosphate dehydrogenase

### Statistical analysis

Graph Pad Prism 9.3.1 was used for conducting the statistical analysis. Data normality was tested using Shapiro-Wilk test. Parametric data were analyzed using One-way ANOVA and Tukey’s post-hoc test, with the results reported as mean ± SEM. Whereas Kruskal-Wallis and Dunn’s tests were employed for non-parametric histopathological scores, presented as median and range. A p-value lower than 0.05 was considered as statistically significant.

A sample size of *n* = 8 animals per group was selected based on previous studies using similar HFD/STZ-induced DN models, which reported statistically detectable biochemical, molecular, and histopathological differences with comparable group sizes. Although no formal a priori power calculation was performed, this sample size is consistent with common practice in preclinical DN research [32, 33].

Given the large number of endpoints analyzed across metabolic, biochemical, histological, and molecular parameters, the possibility of type I error inflation cannot be fully excluded and is acknowledged as a limitation of the study. However, the consistency of directional changes across independent outcome measures supports the robustness of the observed effects.

## Results

### Effect of silymarin on renal function and injury markers in diabetic nephropathy

**Fig. 1:**
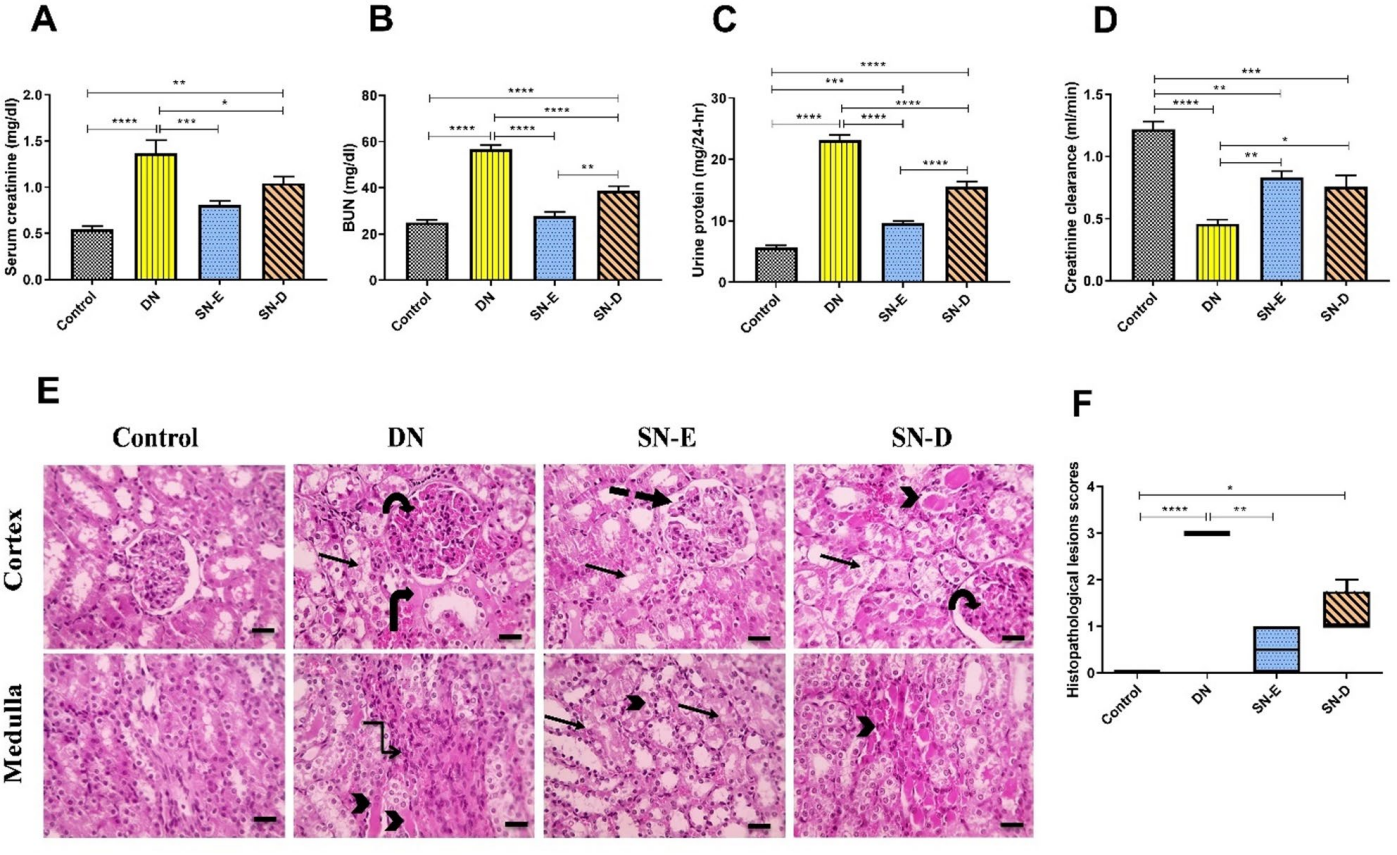
Effect of silymarin on renal function and histopathological alterations in diabetic nephropathy. Renal functions were assessed by measuring serum levels of **A**: serum creatinine, **B**: blood urea nitrogen (BUN), **C**: urine protein and **D**: creatinine clearance. DN: diabetic nephropathy, SN-E: silymarin early intervention group, SN-D: silymarin delayed treatment group. Data are represented as Mean $\:\pm\:$ SEM (*n* = 8 per group) (*p$\:\:<$ 0.05, **p$\:\:<$ 0.01, ***p$\:\:<\:$0.001, ****p$\:\:<$ 0.0001). **E**: Microscopic images of hematoxylin and eosin (H&E)-stained renal sections showing normal cortex and medulla in the control group, severe pathological changes in the cortex and medulla in DN group, significant improvement in the histopathological picture in SN-E group, and moderate improvement in the histopathological picture in SN-D group. Black arrows: diffuse tubular hydropic degeneration, dashed arrows: edematous Bowman’s capsule, curved arrow: interstitial edema, black arrowheads: cast formation, circular arrows: congested glomeruli, elbow arrows: congested inter-tubular blood vessels. X: 400, scale bar = 50 micrometer. **F**: Renal histopathological changes were assessed semi-quantitatively and given scores from 0–3, where 0 is normal, 1 is mild, 2 is moderate, and 3 is severe. DN: diabetic nephropathy, SN-E: silymarin early intervention group, SN-D: silymarin delayed treatment group. Data are represented as median and range (*n* = 8 per group) (*p$\:\:<$ 0.05, ***p$\:\:<\:$0.01, ****p$\:\:<$ 0.0001)

Renal functions were assessed by measuring serum creatinine, BUN, 24-hr-urine protein, and creatinine clearance. As shown in Fig. 1A, B, C, and D, untreated diabetic rats in the DN group exhibited significantly elevated levels of serum creatinine, BUN, and 24-hr-urine protein and a significant reduction of creatinine clearance with regard to the control group (*p* < 0.0001). Administration of silymarin to diabetic rats as an early intervention (SN-E group) or a delayed treatment (SN-D group) resulted in significantly reduced serum creatinine by 41% (*p* < 0.001) and 23.7% (*p* < 0.05), BUN by 50.7% (*p* < 0.0001) and 32.2% (*p* < 0.0001), and 24-hr-urine protein by 58.3% (*p* < 0.0001) and 33.1% (*p* < 0.0001), respectively, as well as a significantly elevated creatinine clearance by 82% (*p* < 0.01) and 65.9% (*p* < 0.05) with regard to the DN group. Notably, a significant difference existed within SN-E and SN-D groups regarding BUN (*p* < 0.01) and 24-hr-urine protein (*p* < 0.0001). Compared with control group, SN-E group demonstrated non-significant differences regarding serum creatinine and BUN whereas 24-hr-urine protein was still significantly elevated (*p* < 0.001) and creatinine clearance was still significantly reduced (*p* < 0.01). Meanwhile, the SN-D group demonstrated a significant difference in all measured biomarkers relative to control group, where serum creatinine (*p* < 0.01), BUN (*p* < 0.0001), and 24-hr-urine protein (*p* < 0.0001) were still markedly elevated while creatinine clearance (*p* < 0.001) was still significantly reduced.

### Effect of silymarin on renal histopathological alterations in diabetic nephropathy

The histopathological changes in kidney tissue were examined in renal tissue segments stained with H&E. The kidney architecture in control group’s microscopic images was normal, as were the glomerular and tubular structures. Severe histopathological lesions were observed in the DN group in both cortex and medulla, including diffuse tubular hydropic degeneration, hyaline casts, interstitial edema, congested glomeruli, and congested inter-tubular blood vessels. SN-E group showed a significantly improved histopathological picture with regard to the DN group (*p* < 0.01) with mild tubular hydropic degeneration and edematous Bowman’s capsule. While the SN-D group showed a moderate improvement in histopathological lesions including moderate tubular hydropic degeneration, hyaline casts, and congested glomeruli with no significant difference compared to the DN group. In addition, the histopathological lesions exhibited non-significant variation between SN-E and control groups, however, significantly higher in SN-D group (*p* < 0.05) (Fig. 1E and F).

### Effect of silymarin on glycemic control and insulin resistance in diabetic rats

**Fig. 2:**
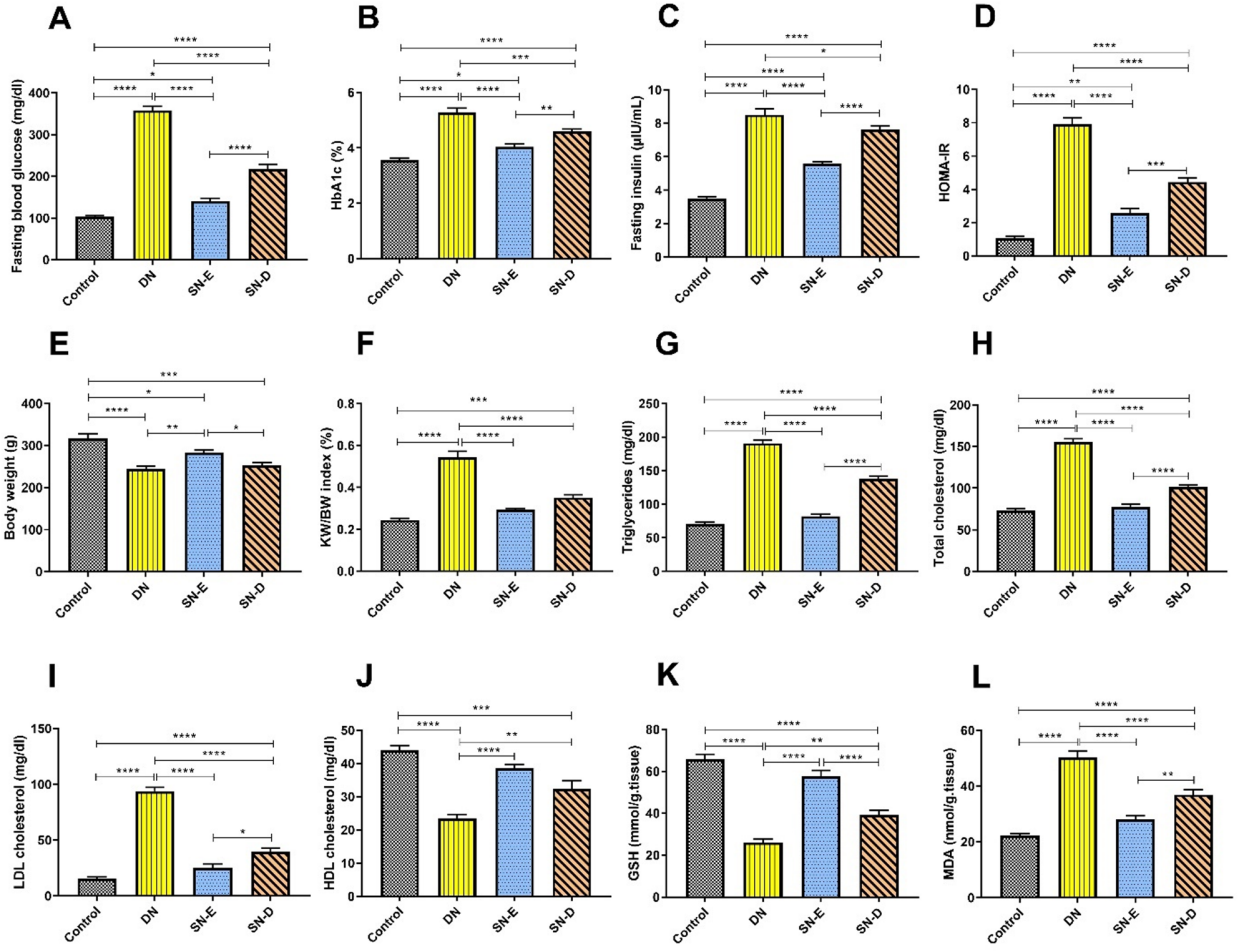
Effect of silymarin on metabolic disturbances and oxidative stress in diabetic rats. The effect of silymarin on hyperglycemia, long term glycemic control, insulin resistance and body weight changes was assessed by measuring fasting blood glucose level (**A**), glycated hemoglobin (HbA1c) % (**B**), fasting insulin level (**C**), HOMA-IR (**D**) and body weight (**E**). Renal hypertrophy was assessed by measuring kidney weight/body weight (KW/BW) index (**F**). The effect of silymarin on lipid profile was assessed by measuring serum levels of triglycerides (**G**), total cholesterol (**H**), low-density lipoprotein (LDL) cholesterol (**I**), and high-density lipoprotein (HDL) cholesterol (**J**). Oxidative stress was assessed by measuring levels of reduced glutathione (GSH) (**K**) and malondialdehyde (MDA) (**L**) spectrophotometrically in renal tissue. DN: diabetic nephropathy, SN-E: silymarin early intervention group, SN-D: silymarin delayed treatment group. Data are represented as Mean $\:\pm\:$ SEM (*n* = 8 per group) (*p$\:\:<$ 0.05, **p$\:\:<$ 0.01, ***p$\:\:<$ 0.001, ****p$\:\:<$ 0.0001)

As shown in Fig. 2A, B, C and D, untreated diabetic rats in the DN group revealed substantially higher fasting blood glucose levels, HbA1c percentage, fasting insulin levels and HOMA-IR (*p* < 0.0001) relative to the control group. Administration of silymarin as an early intervention significantly reduced the elevated fasting blood glucose, HbA1c percentage, fasting insulin levels and HOMA-IR in SN-E group relative to DN group (*p* < 0.0001). Whereas delayed silymarin treatment in SN-D group significantly reduced fasting blood glucose (*p* < 0.0001), HbA1c percentage (*p* < 0.001), fasting insulin levels (*p* < 0.05) and HOMA-IR (*p* < 0.0001), relative to DN group. Notably, fasting blood glucose, HbA1c percentage, fasting insulin levels and HOMA-IR were more significantly reduced in the SN-E group by 60.6%, 23.6%, 34.4% and 67.5% compared to 39.2%, 13%, 10.4% and 44% decrease in the SN-D group (*p* < 0.0001, *p* < 0.01, *p* < 0.0001, and *p* < 0.001, respectively). However, fasting blood glucose, HbA1c percentage, fasting insulin levels and HOMA-IR were still significantly elevated in SN-E group (*p* < 0.05, *p* < 0.05, *p* < 0.0001, and *p* < 0.01, respectively) and SN-D group (*p* < 0.0001) with respect to control group.

### Effect of silymarin on metabolic disturbances in diabetic rats

As shown in Fig. 2E, diabetic rats exhibited significant alterations in body weight, demonstrating a 1.3-fold reduction in body weight compared with the control group (*p* < 0.0001). Silymarin-treated groups demonstrated partial improvement in body weight changes, increasing body weight by 13.9% in SN-E group (*p* < 0.01) and non-significantly in SN-D group, compared to DN group. Notably, early intervention in SN-E group showed greater preservation of body weight compared with delayed treatment in SN-D group (*p* < 0.05). Figure 2F revealed a significantly elevated kidney weight/body weight (KW/BW) index in DN group relative to the control group (*p* < 0.0001) which indicates significant renal hypertrophy in untreated diabetic rats. A significant reduction in KW/BW index was observed in SN-E group by 45.7% (*p* < 0.0001) and in SN-D group by 35% (*p* < 0.0001) as compared to DN group. However, KW/BW index was still substantially increased in SN-D versus control group (*p* < 0.001) while non-significant variation was found between SN-E and control groups.

As demonstrated by Fig. 2G, H, I, and J, the lipid profile biomarkers were markedly improved when diabetic rats received silymarin as an early or delayed treatment. DN group demonstrated significantly increased serum triglycerides, total cholesterol, and LDL cholesterol levels and significantly decreased HDL cholesterol level relative to the control group (*p* < 0.0001). Administering silymarin to diabetic rats in SN-E and SN-D groups resulted in a substantial decrease in serum triglycerides by 57% and 27.7%, total cholesterol by 50.2% and 35.9%, and LDL cholesterol by 73.3% and 57.8% (*p* < 0.0001) and a significant elevation in HDL cholesterol levels by 64.5% (*p* < 0.0001) and 38.2% (*p* < 0.01), respectively, relative to the DN group. Notably, a significant difference existed between SN-E and SN-D groups regarding serum triglycerides (*p* < 0.0001), total cholesterol (*p* < 0.0001), and LDL cholesterol (*p* < 0.05) levels. With respect to the control group, the SN-E group revealed no significant difference concerning all the studied lipid biomarkers whereas the SN-D group revealed significantly higher levels of serum triglycerides (*p* < 0.0001), total cholesterol (*p* < 0.0001) and LDL cholesterol (*p* < 0.0001) levels and a significantly lower HDL cholesterol level (*p* < 0.001).

### Effect of silymarin on oxidative stress and lipid peroxidation in diabetic kidneys

As demonstrated by Fig. 2K, renal GSH level was substantially lower in DN group relative to control group (*p* < 0.0001), which indicates that diabetic rats were under a considerably high level of oxidative stress. Silymarin administration to diabetic rats in both SN-E and SN-D groups caused a substantial rise in GSH level by 54.7% (*p* < 0.0001) and 33.5% (*p* < 0.01), respectively, relative to DN group. The SN-E group revealed remarkably increased levels of GSH relative to SN-D group (*p* < 0.0001). In addition, SN-E group exhibited a non-significant difference in renal GSH level with respect to the control group, whereas the GSH level was still markedly lower in SN-D group relative to control group (*p* < 0.0001).

Renal MDA levels were remarkably higher in the DN group than the control group (*p* < 0.0001), revealing a considerable rise in lipid peroxidation (Fig. 2L). Silymarin administration to diabetic rats in both SN-E and SN-D groups caused substantially reduced MDA levels by 43.9% and 26.4%, respectively, relative to DN group (*p* < 0.0001). The SN-E group displayed substantially lower levels of MDA relative to the SN-D group (*p* < 0.01). Additionally, SN-E group exhibited non-significant difference in renal MDA level with respect to the control group, whereas the MDA level was still markedly elevated in SN-D versus control group (*p* < 0.0001).

### Effect of silymarin on renal miRNA-223/NLRP3/caspase-1 gene expression

**Fig. 3:**
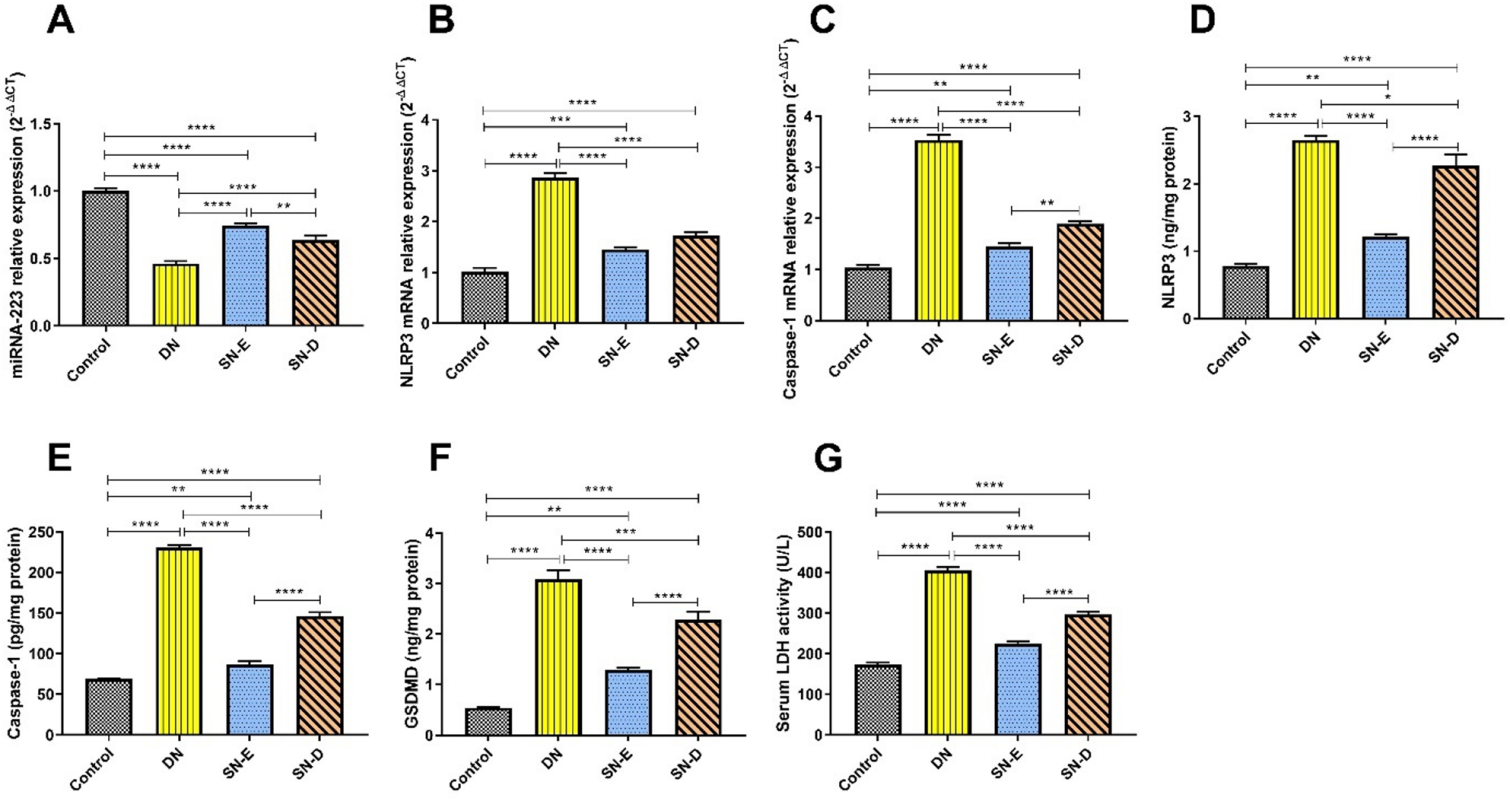
Effect of silymarin on miRNA-223 expression and NLRP3/Caspase-1/GSDMD axis. Quantitative reverse transcription polymerase chain reaction (RT-qPCR) was used to measure relative expression of miRNA-223 (**A**), NLRP3 (**B**), and caspase-1 (**C**) mRNA relative expression in renal tissue. Renal protein levels of NLRP3 (**D**), caspase-1 (**E**), and GSDMD (**F**) were measured using enzyme-linked immunosorbent assay (ELISA) technique. Serum LDH activity (**G**) was measured spectrophotometrically. DN: diabetic nephropathy, SN-E: silymarin early intervention group, SN-D: silymarin delayed treatment group, NLRP3: NOD-like receptor family pyrin domain-containing 3, GSDMD: gasdermin D, LDH: lactate dehydrogenase. Data are represented as Mean $\:\pm\:$ SEM (*n* = 8 per group) (*p$\:\:<$ 0.05, **p$\:\:<$ 0.01, ***p$\:\:<$ 0.001, ****p$\:\:<$ 0.0001)

As demonstrated by Fig. 3A, the DN group revealed a significantly decreased miRNA-223 relative expression with regard to control group (*p* < 0.0001). SN-E and SN-D groups exhibited a significant elevation in miRNA-223 relative expression, with regard to the DN group (*p* < 0.0001), with a more significantly higher miRNA-223 relative expression in the SN-E group (61.2%) compared to 42.1% in the SN-D group (*p* < 0.01). However, miRNA-223 relative expression was still significantly lower in either the SN-E or SN-D group with respect to the control group (*p* < 0.0001).

Figures 3B and C demonstrated a significant elevation in NLRP3 and caspase-1 mRNA relative expression in the DN group with regard to the control group (*p* < 0.0001). When diabetic rats were given silymarin in the early intervention (SN-E) group or the delayed treatment (SN-D) group, a significant decrease was observed in NLRP3 by 49.3% and 40%, and caspase-1 by 59.1% and 46.5%, respectively, when compared to DN group (*p* < 0.0001). Notably, a remarkable difference was observed between SN-E and SN-D groups regarding caspase-1 (*p* < 0.01) mRNA relative expression. Compared with control group, NLRP3 and caspase-1 mRNA relative expressions were still significantly elevated in both the SN-E group (*p* < 0.001) and (*p* < 0.01), respectively, as well as the SN-D group (*p* < 0.0001).

### Effect of silymarin on NLRP3 inflammasome activation and pyroptosis-related signaling in diabetic kidneys

As shown in Fig. 3D, E, F and G, renal expression of NLRP3, caspase-1, and GSDMD as well as serum LDH activity were markedly elevated in the DN group compared with the control group (*p* < 0.0001), indicating activation of inflammasome-related signaling. Treatment with silymarin resulted in a reduction of these markers in both experimental groups, with a more pronounced effect observed in the SN-E group compared with SN-D. In the SN-E group, renal NLRP3, caspase-1, and GSDMD levels as well as serum LDH activity were significantly reduced by 54%, 62.3%, 57.9% and 44.9%, respectively, relative to the DN group (*p* < 0.0001), whereas the SN-D group showed a moderate but still significant reduction across the same markers, reducing NLRP3, caspase-1, GSDMD levels and serum LDH activity by 14.3% (*p* < 0.05), 36.7% (*p* < 0.0001), 26.1% (*p* < 0.001) and 27.1% (*p* < 0.0001), respectively, relative to DN group. Consistently, renal expression levels of NLRP3, caspase-1, and GSDMD along with serum LDH activity were lower in the SN-E group compared with the SN-D group (*p* < 0.0001), indicating a greater response to early intervention. However, neither intervention fully restored these markers to control levels, as both SN-E and SN-D groups remained significantly higher than the control group, with a more persistent elevation observed in the SN-D group.

### Effect of silymarin on NF-κB (p65) signaling and inflammatory cytokines in diabetic kidneys

**Fig. 4:**
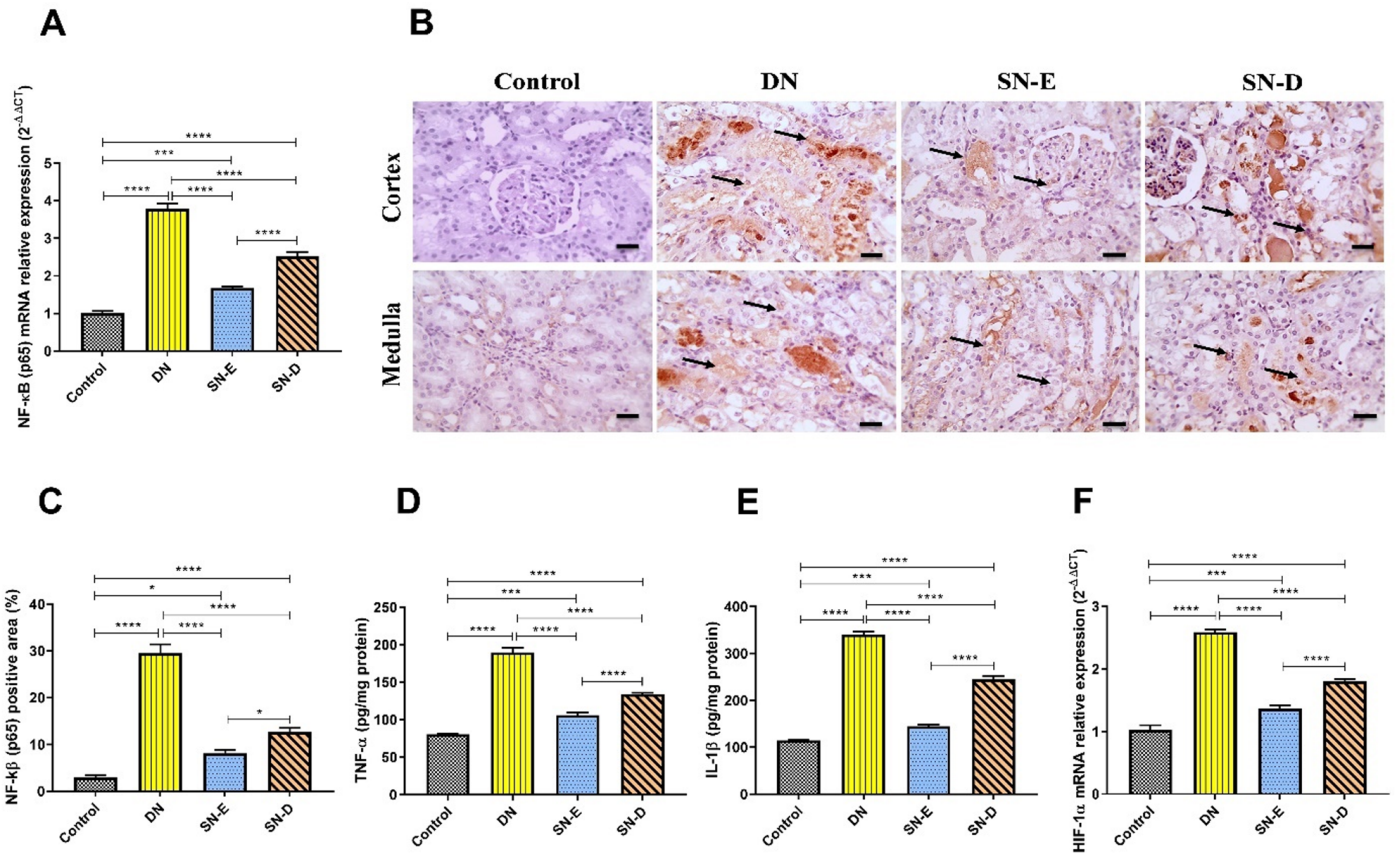
Effect of silymarin on NF-κB (p65) signaling, inflammatory cytokines, and hypoxia. **A**: Quantitative reverse transcription polymerase chain reaction (RT-qPCR) was used to measure relative expression of NF-κB (p65) in renal tissue. **B**: Microscopic images of immunostained renal sections against NF-κB (p65) showing negative staining in the cortex and medulla in the control group, excess brown tubular staining in the cortex and medulla in the DN group, decreased brown tubular staining in the cortex and medulla in SN-E group and SN-D group. Black arrows refer to nuclear staining. IHC counterstained with Mayer’s hematoxylin. X: 400, scale bar = 50 micrometer. **C**: Immunostaining of NF-κB (p65) was assessed quantitatively through the area of positive expression. Renal tissue protein levels of TNF-α (**D**) and IL-1β (**E**) were measured using enzyme-linked immunosorbent assay (ELISA) technique. **F**: Quantitative real-time polymerase chain reaction (qRT-PCR) was used to measure relative expression of HIF-1α in renal tissue. DN: diabetic nephropathy, SN-E: silymarin early intervention group, SN-D: silymarin delayed treatment group, NF-κB (p65): nuclear factor-kappa B (p65), TNF-α: tumor necrosis factor-alpha, IL-1β: interleukin-1 beta, HIF-1α: hypoxia-inducible factor-1 alpha. Data are represented as Mean $\:\pm\:$ SEM (*n* = 8 per group) (*p$\:\:<$ 0.05, ***p$\:\:<$ 0.001, ****p$\:\:<$ 0.0001)

In this study, renal inflammation was assessed by measuring both gene and protein expression of NF-κB (p65) as well as renal tissue levels of TNF-α and IL-1β. Figure 4A demonstrated a significant elevation in NF-κB (p65) mRNA relative expression in the DN group with regard to the control group (*p* < 0.0001). When diabetic rats were given silymarin in the early intervention (SN-E) group or the delayed treatment (SN-D) group, a significant decrease was observed in NF-κB (p65) by 55.7% and 33.1%, respectively, when compared to DN group (*p* < 0.0001). Notably, a remarkable difference was observed between SN-E and SN-D groups regarding NF-κB (p65) (*p* < 0.0001) mRNA relative expression. Compared with control group, NF-κB (p65) mRNA relative expression was still significantly elevated in both the SN-E group (*p* < 0.001) and the SN-D group (*p* < 0.0001).

The protein expression of NF-κB (p65) was evaluated in renal tissue using immunohistochemistry. The microscopic images of immunostained renal sections showed no brown staining in either the cortex or medulla in the control group, indicating the absence of NF-κB (p65) expression. In contrast, the DN group exhibited excess brown tubular staining in both the cortex and medulla, indicating elevated expression of NF-κB (p65). Additionally, there was excess brown nuclear staining in tubules, indicating elevated nuclear expression of activated NF-κB (p65). NF-κB (p65) expression was considerably elevated in the DN group relative to control group (*p* < 0.0001). SN-E and SN-D groups exhibited a significantly reduced NF-κB (p65) expression relative to DN group (*p* < 0.0001). Meanwhile, the SN-E group revealed more significant reduction as compared to SN-D group (*p* < 0.05). In comparison with control group, NF-κB (p65) expression was still markedly elevated in either SN-E (*p* < 0.05) or SN-D (*p* < 0.0001) group (Fig. 4B and C).

Figures 4D and E revealed significantly elevated TNF-α and IL-1β in DN group, respectively, with regard to control group (*p* < 0.0001). Diabetic rats administered silymarin in both SN-E and SN-D groups displayed a remarkable reduction in TNF-α by 44% and 29.1%, and IL-1β by 57.5% and 28%, respectively, with regard to DN group (*p* < 0.0001). Additionally, the SN-E group showed significantly lower levels of TNF-α and IL-1β in comparison with the SN-D group (*p* < 0.0001). However, TNF-α and IL-1β were still significantly elevated in the SN-E (*p* < 0.001) and SN-D (*p* < 0.0001) groups relative to the control group.

### Effect of silymarin on renal tissue hypoxia in diabetic kidneys

As shown in Fig. 4F, a significant elevation was obvious in HIF-1α mRNA relative expression in DN group versus control group (*p* < 0.0001). Administration of silymarin to diabetic rats significantly reduced HIF-1α mRNA relative expression in SN-E group by 47% and in the SN-D group by 30.2% relative to DN group (*p* < 0.0001). Significantly different HIF-1α mRNA relative expressions were found in SN-E and SN-D groups (*p* < 0.0001). However, both SN-E and SN-D groups still showed significantly elevated HIF-1α mRNA relative expression (*p* < 0.001) and (*p* < 0.0001), respectively, relative to control group.

### Effect of silymarin on renal fibrosis and extracellular matrix deposition

**Fig. 5:**
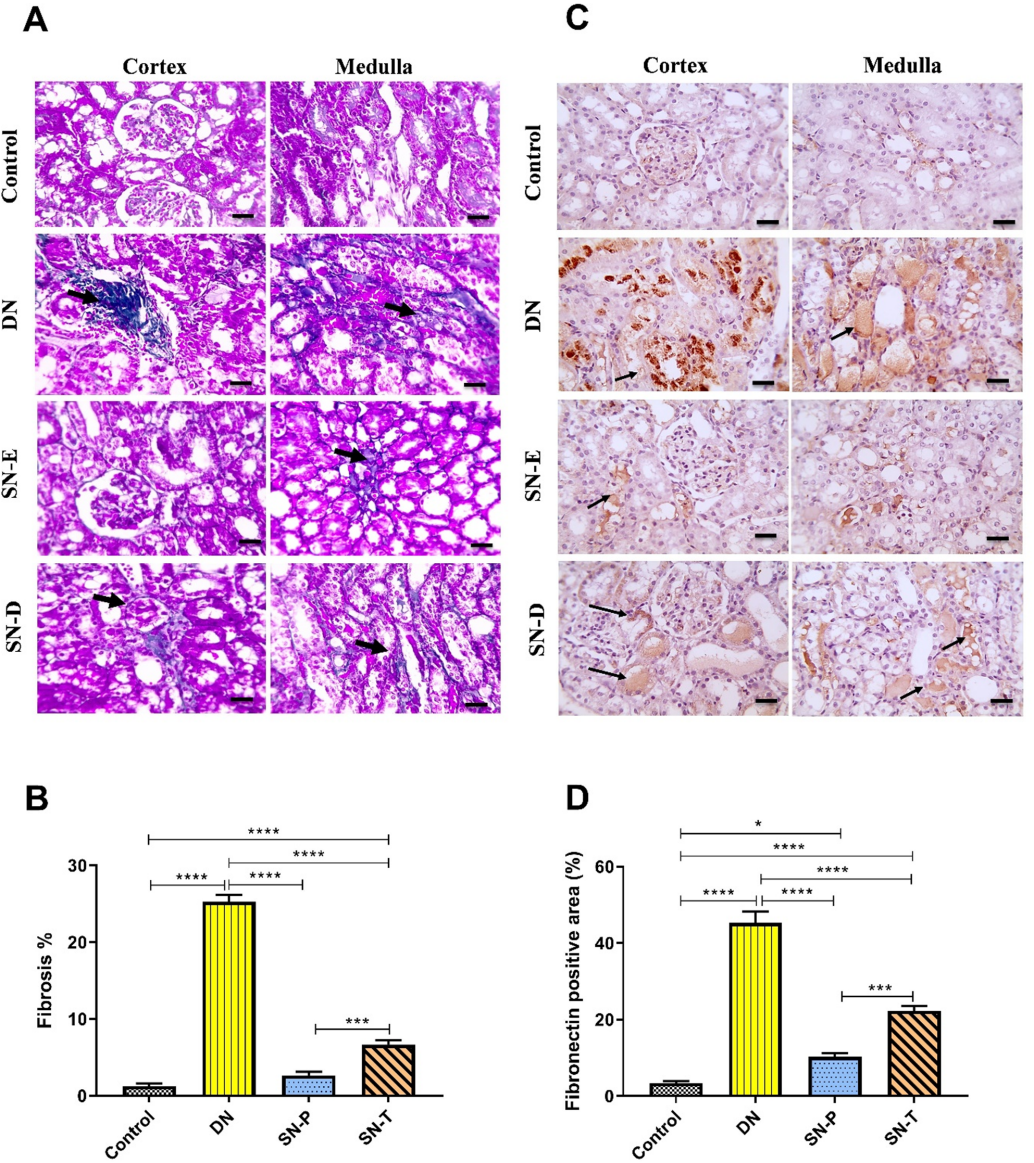
Effect of silymarin on renal fibrosis and extracellular matrix deposition. **A**: Microscopic images of Masson’s trichrome-stained renal sections showing no collagen deposition in the cortex or medulla in the control group, excess bluish-stained collagen deposition in the cortex and medulla in the DN group, and considerably decreased collagen deposition in the cortex and medulla in SN-E and SN-D groups. Black arrows refer to collagen deposition. X: 400, scale bar = 50 micrometer. **B**: Fibrosis percentage was assessed quantitatively through Masson’s-positive area percentage. **C**: Microscopic images of immunostained renal sections against fibronectin showing negative staining in the cortex and medulla in the control group, excess brown tubular staining in the cortex and medulla in DN group, and markedly decreased positive brown tubular staining in the cortex and medulla in SN-E and SN-D groups. Black arrows refer to fibronectin deposition. Immunohistochemistry counterstained with Mayer’s hematoxylin. X: 400, scale bar = 50 micrometer. **D**: Immunostaining of fibronectin was assessed quantitatively through the area of positive expression. DN: diabetic nephropathy, SN-E: silymarin early intervention group, SN-D: silymarin delayed treatment group. Data are represented as Mean $\:\pm\:$ SEM (*n* = 8 per group) (*p$\:\:<$ 0.05, ***p$\:\:<$ 0.001, ****p$\:\:<$ 0.0001)

On microscopic examination, there was no extra collagen deposited in the cortex or medulla of kidney tissue segments stained by Masson trichrome staining in control group. The renal cortex and medulla of the DN group displayed an excess of bluish-stained collagen deposition, indicating severe fibrosis. Considerably reduced bluish-stained collagen deposition was seen in the SN-E and SN-D groups and the fibrosis percentage was reduced significantly (*p* < 0.0001) with respect to the DN group. Additionally, the fibrosis percentage was more significantly reduced in the SN-E group relative to SN-D group (*p* < 0.001). However, fibrosis percentage was still significantly higher in SN-D (*p* < 0.0001) group relative to control group (Fig. 5A and B).

Renal fibrosis was further assessed by immunohistochemical evaluation of fibronectin deposition in immunostained renal sections. The microscopic images of immunostained kidney tissue segments against fibronectin didn’t exhibit any extra brown staining in the control group’s cortex and medulla. In contrast, the DN group displayed excessive brown tubular staining, signifying considerably more fibronectin deposition relative to control group (*p* < 0.0001). Brown tubular staining was noticeably reduced in both the cortex and medulla of SN-E and SN-D groups, and fibronectin deposition percentage was significantly lower relative to the DN group (*p* < 0.0001). Moreover, the fibronectin deposition was more significantly decreased in SN-E group with regard to SN-D group (*p* < 0.001). The fibronectin deposition was still significantly higher in SN-E (*p* < 0.05) group and SN-D group (*p* < 0.0001) relative to the control group (Fig. 5C and D).

## Discussion

Diabetic nephropathy represents a progressive metabolic and inflammatory disorder characterized by the convergence of hyperglycemia-induced oxidative stress, dysregulated lipid metabolism, chronic inflammation, hypoxic injury, and fibrotic remodeling [34]. Despite the availability of current anti-diabetic therapies, their efficacy in preventing the progression of DN to ESRD remains limited [35]. Consequently, there is a critical need to identify novel therapeutic strategies that can effectively target the underlying pathogenic mechanisms, particularly inflammation-driven renal injury, while preserving kidney function. In this context, the present study was designed to evaluate the renoprotective effects of silymarin as early and delayed treatment interventions in a high-fat diet/streptozotocin (HFD/STZ)-induced model of diabetic nephropathy, with particular focus on its association with modulation of the miRNA-223/NLRP3/caspase-1/GSDMD axis and pyroptosis-associated signaling, in addition to its effects on oxidative stress, hypoxia, inflammation, and fibrotic responses.

The HFD/STZ model employed in this study successfully recapitulated key features of type 2 diabetes–associated DN by combining insulin resistance with partial β-cell dysfunction [36]. The elevated insulin levels and HOMA-IR observed in diabetic rats confirmed the state of insulin resistance with compensatory hyperinsulinemia in the HFD/STZ model. DN establishment in this model was evidenced by marked microalbuminuria, as reflected by increased 24-hour urinary protein excretion, alongside elevated serum creatinine and BUN levels and reduced creatinine clearance, confirming renal functional impairment [37]. Histopathological examination further demonstrated characteristic diabetic renal injury, including tubular degeneration, glomerular congestion, and interstitial alterations. These findings are consistent with previously established experimental models of DN [32, 38].

Silymarin administration significantly improved renal function parameters and ameliorated structural kidney damage in diabetic rats. These findings support the previously reported ameliorative effects of silymarin in DN [39]. Notably, early intervention exerted more pronounced effects than delayed treatment, restoring several parameters toward near-normal levels. This suggests that early modulation of pathogenic pathways confers superior renoprotection compared to late intervention after disease establishment.

Chronic hyperglycemia and insulin resistance are central contributors to DN progression. Accordingly, assessment of fasting glucose and insulin levels, long-term glycemic control markers such as HbA1c and insulin resistance indices including HOMA-IR may provide additional insight into metabolic dysregulation associated with diabetic renal injury. Metabolic disturbances were observed in diabetic rats, as evidenced by persistent hyperglycemia and insulin resistance with compensatory hyperinsulinemia, decreased body weight, and prominent renal hypertrophy which reflects chronic hyperglycemia-induced hyperfiltration [40, 41].

Notably, silymarin significantly improved glycemic control and insulin resistance as evidenced by reduced fasting blood glucose levels, HbA1c %, fasting insulin levels, and HOMA-IR. Silymarin also restored body weight and KW/BW index and attenuated renal hypertrophy, in line with earlier studies [42–45]. However, it is important to note that normoglycemia and restoration of insulin sensitivity were not fully achieved in either the early or delayed treatment groups, indicating that the renoprotective effects of silymarin may not be solely attributable to glycemic control. This observation is consistent with the findings reported by Stolf, et al. in an earlier study [46]. Given the well-established relationship between hyperglycemia and downstream oxidative and inflammatory cascades, part of the observed renoprotective effects of silymarin may be attributable to improvements in metabolic status. Nevertheless, the concurrent modulation of inflammatory and inflammasome-associated markers suggests that silymarin may also exert glucose-independent renoprotective actions through direct attenuation of inflammatory and pyroptotic signaling pathways, rather than acting solely via metabolic correction. However, the relative contribution of glucose-dependent versus direct anti-inflammatory mechanisms could not be definitively distinguished within the scope of the present study.

Dyslipidemia, a hallmark of type 2 diabetes, was also evident in STZ-induced diabetic rats, with elevated triglycerides, total cholesterol, and LDL levels and reduced HDL levels, consistent with insulin resistance-driven alterations in lipid metabolism [47–50]. Silymarin markedly improved the lipid profile, particularly with early intervention, suggesting an additional contribution of lipid-lowering effects to its renoprotective action. These results agree with El-Far, et al. findings in diabetic rats [51], Ebrahimpour-Koujan, et al. findings in T2DM patients [52] and with the findings obtained by Marin, et al. in both non-alcoholic steatohepatitis mice model and human hepatocytes [53].

At the mechanistic level, inflammation represents a central driver of diabetic nephropathy progression. NF-κB functions as a key upstream regulator, promoting the transcription of pro-inflammatory cytokines and providing the priming signal required for NLRP3 inflammasome activation, without which full inflammasome activation cannot be achieved [54–56]. In the present study, diabetic rats exhibited marked NF-κB activation, as evidenced by increased p65 expression and nuclear localization, accompanied by elevated TNF-α and IL-1β levels, indicating an amplified inflammatory response. This inflammatory milieu was associated with increased expression of NLRP3, caspase-1, and GSDMD, suggesting activation of inflammasome-related signaling pathways. While these markers are widely associated with pyroptotic cell death, it should be noted that direct detection of cleaved caspase-1 or the active GSDMD-N fragment was not performed. Therefore, the involvement of pyroptosis is inferred based on established molecular signatures rather than directly demonstrated.

Importantly, the observed elevation in LDH activity further supports the presence of cellular membrane damage and lytic forms of cell death in diabetic kidneys. Although LDH release is not specific to pyroptosis, it is commonly used as a marker of membrane rupture associated with GSDMD-mediated membrane permeabilization and inflammasome-related cell lysis [57].

Silymarin attenuated NF-κB activation and reduced the expression of downstream inflammatory mediators and inflammasome-associated markers including NLRP3, caspase-1, IL-1β and GSDMD, along with a significant reduction in LDH activity. These findings collectively suggest that silymarin mitigates inflammation-driven cellular injury and modulates signaling pathways associated with inflammasome activation and pyroptosis-related signaling.

Importantly, the present findings are consistent with previously reported evidence supporting a regulatory relationship between miRNA-223 and NLRP3 inflammasome activity. Diabetic rats exhibited significant downregulation of renal miRNA-223 expression together with enhanced inflammasome signaling, consistent with previous reports linking miRNA-223 deficiency to enhanced inflammasome activation [58–61]. Silymarin markedly upregulated miRNA-223 expression, accompanied by suppression of NLRP3, caspase-1, IL-1β, and GSDMD. These findings suggest a potential association between restoration of miRNA-223 expression and suppression of inflammasome-related signaling following silymarin treatment. These observations are consistent with those reported by Tang, et al. who demonstrated that silibinin, the major active component of silymarin, alleviated acute liver injury through induction of miRNA-223 expression [62].

However, it should also be considered that silymarin is a pleiotropic compound with well-documented antioxidant, anti-inflammatory, antifibrotic, and metabolic effects [63]. Therefore, the observed renoprotective actions are unlikely to be attributable exclusively to modulation of the miRNA-223/NLRP3 axis. Rather, the present findings suggest that regulation of miRNA-223 and inflammasome-related signaling may represent one component within a broader network of interconnected protective mechanisms involving attenuation of oxidative stress, inflammatory signaling, metabolic dysregulation, hypoxia, and fibrosis. Accordingly, while the coordinated changes observed in the current study support a potential association between miRNA-223 modulation and suppression of inflammasome-related pathways, further mechanistic studies are required to define the relative contribution and specificity of this pathway in mediating the effects of silymarin in diabetic nephropathy.

Oxidative stress represents another major contributor to renal injury, as evidenced by elevated MDA levels and depleted GSH in diabetic kidneys. These alterations reflect enhanced lipid peroxidation and impaired antioxidant defense systems, which are known to promote inflammasome activation and tissue damage [64]. These findings are in agreement with those of Awad, et al. and Mi, et al. in DN animal models [65, 66]. Silymarin, particularly in the early intervention regimen, effectively restored redox balance by reducing MDA and increasing GSH levels, consistent with its well-established antioxidant properties [45, 67]. This redox modulation likely suppresses NLRP3 activation and subsequent pyroptosis, in line with the established role of ROS as a secondary signal driving inflammasome assembly in DN.

Hypoxia, defined by insufficient oxygen availability, emerged as a critical contributor to diabetic renal injury, as evidenced by the marked upregulation of HIF-1α in diabetic kidney tissue [68]. Beyond serving as a hypoxic marker, HIF-1α acts as a central transcriptional regulator that integrates hypoxic and inflammatory signaling. Mechanistically, HIF-1α can potentiate inflammatory responses through functional interaction with NF-κB, enhancing its nuclear translocation and transcriptional activity. In parallel, sustained NF-κB activation and the inflammatory milieu can further augment HIF-1α expression, establishing a bidirectional regulatory loop between hypoxia and inflammation [11]. Importantly, HIF-1α activation also drives the expression of pro-fibrotic mediators and promotes extracellular matrix (ECM) accumulation, thereby contributing to progressive renal fibrosis [69]. Moreover, hypoxia exacerbates oxidative stress by increasing ROS generation, while oxidative stress, in turn, stabilizes HIF-1α and reinforces its downstream effects [70]. Silymarin significantly reduced HIF-1α expression, suggesting an improvement in renal oxygen homeostasis and interruption of hypoxia-driven injury.

**Fig. 6:**
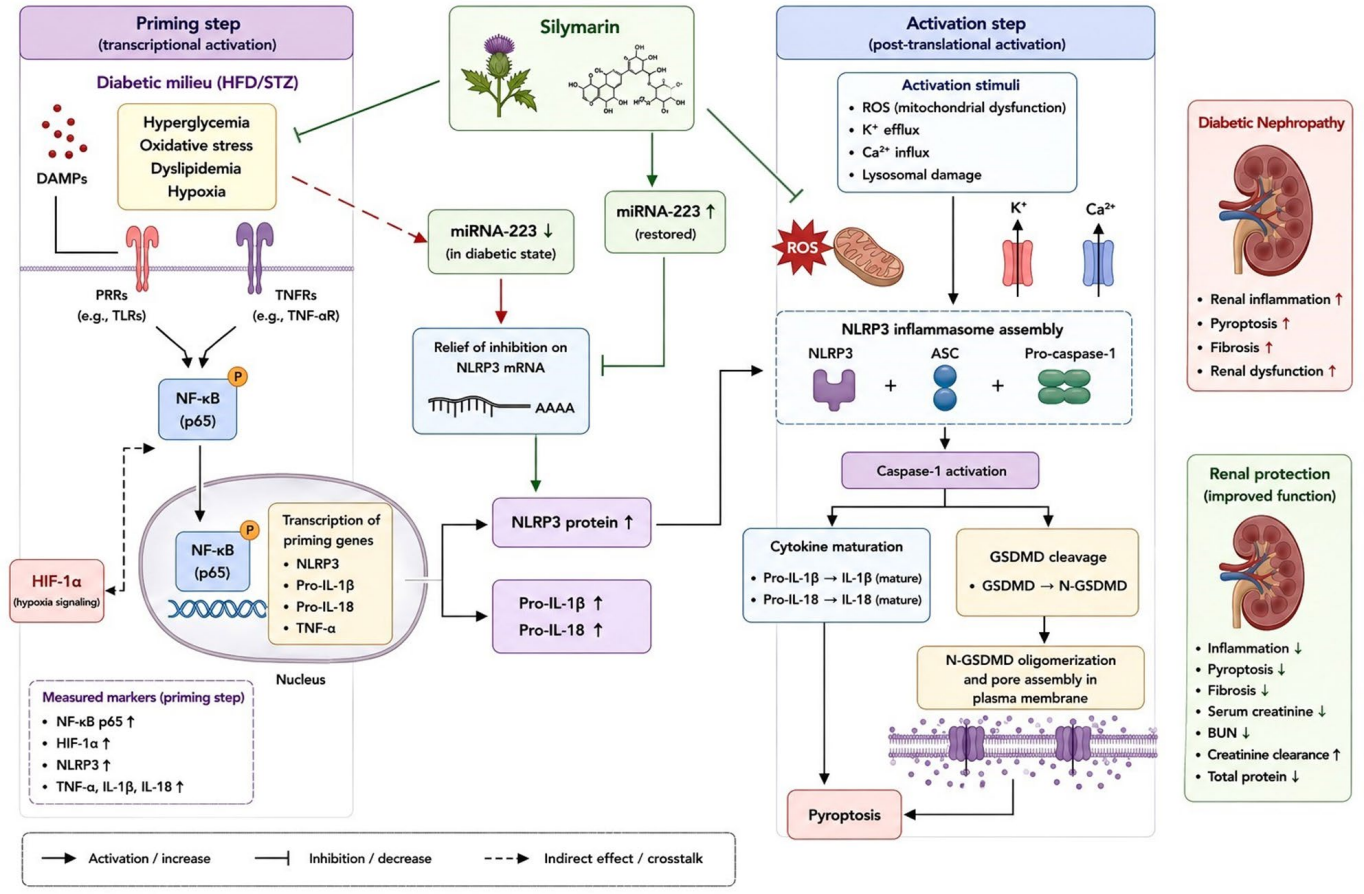
Proposed mechanism underlying the protective effects of silymarin against HFD/STZ-induced diabetic nephropathy. The schematic figure illustrates the proposed molecular events involved in diabetic nephropathy (DN) and the protective mechanisms mediated by silymarin. In the priming step, the diabetic milieu induced by high-fat diet and streptozotocin (HFD/STZ), characterized by hyperglycemia, oxidative stress, dyslipidemia, and hypoxia, activates PRRs/TLRs and TNFRs, leading to NF-κB (p65) activation and transcriptional priming of NLRP3, pro-IL-1β, pro-IL-18, and TNF-α. HIF-1α signaling may further contribute to inflammatory activation through indirect crosstalk with NF-κB. Reduced miRNA-223 expression in the diabetic state relieves inhibitory regulation on NLRP3, promoting increased NLRP3 expression. In the activation step, mitochondrial dysfunction-induced ROS, K^+^ efflux, Ca^2+^ influx, and lysosomal damage promote NLRP3 inflammasome assembly with ASC and pro-caspase-1, resulting in caspase-1 activation. Activated caspase-1 mediates maturation of IL-1β and IL-18 and induces GSDMD cleavage, leading to membrane pore formation and pyroptosis, which contribute to renal inflammation, fibrosis, renal dysfunction, and progression of DN. Silymarin administration is proposed to attenuate these pathological events through attenuation of diabetes-associated metabolic and inflammatory disturbances and restoration of miRNA-223 expression, thereby limiting NLRP3 inflammasome activation and downstream pyroptotic signaling. These effects contribute to reduced inflammation, pyroptosis, and fibrosis, ultimately improving renal function. DAMPs: damage-associated molecular patterns, PRRs: pattern recognition receptors, TLRs: toll-like receptors, TNFRs: tumor necrosis factor receptors, NF-κB: nuclear factor-kappa B, NLRP3: NOD-like receptor family pyrin domain-containing 3, IL-1β: interleukin-1 beta, IL-18: interleukin-18, TNF-α: tumor necrosis factor-alpha, HIF-1α: hypoxia-inducible factor-1 alpha, ROS: reactive oxygen species, ASC: apoptosis-associated speck-like protein containing a caspase recruitment domain, GSDMD: gasdermin D

Collectively, these findings suggest that oxidative stress, inflammation, and hypoxia do not operate as independent processes, but rather as interconnected drivers that converge on inflammasome activation and fibrotic remodeling in DN. Consistent with these findings, marked renal fibrosis was observed in diabetic rats, as indicated by increased collagen deposition and fibronectin expression. These changes reflect excessive ECM accumulation driven by chronic inflammation, oxidative stress, and hypoxia [71]. Silymarin significantly attenuated fibrotic remodeling, likely through integrated modulation of these upstream processes. This was consistent with an earlier study which reported that silibinin, the primary active component of silymarin, exerted renoprotective effects by inhibiting renal fibrosis and preventing extracellular matrix deposition [72]. The greater efficacy of early intervention compared to delayed treatment further underscores its importance in preventing irreversible structural damage. The proposed mechanisms underlying the protective effects of silymarin against HFD/STZ-induced DN are illustrated in Fig. 6.

Current therapeutic strategies for DN primarily focus on glycemic control, blood pressure regulation, and attenuation of renal inflammation and fibrosis through agents such as renin-angiotensin system inhibitors and sodium-glucose cotransporter-2 (SGLT2) inhibitors. In addition to their metabolic benefits, several of these therapies exert renoprotective effects through modulation of oxidative stress and inflammatory signaling pathways [73]. In this context, the present findings suggest that silymarin may represent a complementary multi-target approach capable of simultaneously modulating metabolic disturbances, oxidative stress, inflammasome-related signaling, hypoxia, and fibrosis. Nevertheless, the current study remains preclinical in nature, and the observed effects should not be interpreted as evidence of equivalence or superiority to established standard-of-care therapies. Further comparative and translational studies are required to define the therapeutic relevance of silymarin in DN.

This study has several limitations that should be acknowledged. First, the proposed involvement of miRNA-223 in regulating NLRP3 inflammasome signaling is based on associative evidence, as functional validation experiments such as miRNA inhibition or overexpression were not performed. Second, pyroptotic cell death was inferred from upstream signaling markers, but key executional readouts, including cleaved caspase-1 (p20) and GSDMD-N, were not directly assessed. Third, inflammasome activation was evaluated indirectly, and assessment of ASC speck formation or oligomerization would have provided stronger evidence of inflammasome assembly; however, this was not performed in the present study. Fourth, the study did not include a standard therapeutic comparator or a non-diabetic silymarin-treated group, which would have strengthened interpretation of the findings. Fifth, only a single dose of silymarin and male animals were used, which may limit generalizability. Sixth, although expanded metabolic profiling was incorporated in this study, the current design does not fully distinguish direct molecular effects of silymarin from those secondary to improved glycemic control. Future studies employing pair-fed or insulin-matched controls may help clarify glucose-dependent versus glucose-independent mechanisms. Finally, although the HFD/STZ model replicates key features of type 2 diabetes-associated DN, it does not fully recapitulate the complexity of human disease. Future studies incorporating longer-duration protocols, additional clinical endpoints, and complementary experimental systems will be important to further evaluate the translational relevance of silymarin in diabetic nephropathy.

## Conclusion

Silymarin demonstrates significant renoprotective effects in experimental diabetic nephropathy, acting through multiple complementary mechanisms. Its beneficial actions are associated with modulation of miRNA-223 expression and concomitant downregulation of the NLRP3/caspase-1/GSDMD signaling axis, suggesting a potential link with reduced inflammasome activation and pyroptosis-related pathways. In parallel, silymarin improves metabolic disturbances, attenuates oxidative stress, alleviates hypoxic signaling, and limits renal fibrotic remodeling, indicating a broad spectrum of disease-modifying effects. Early intervention showed superior efficacy compared to delayed treatment, highlighting the importance of timely therapeutic targeting in diabetic kidney injury. However, the observed association between miRNA-223 and inflammasome signaling remains correlative, and direct mechanistic validation, as well as definitive confirmation of pyroptotic cell death, is still required. Overall, these findings support silymarin as a promising multi-target candidate for diabetic nephropathy and justify further mechanistic and translational studies.

## Data Availability

Data are available from the corresponding author upon reasonable request.

## Abbreviations

DN: Diabetic nephropathy
T2DM: Type 2 diabetes mellitus
ESRD: End-stage renal disease
NF-κB: Nuclear factor-kappa B
NLRP3: NOD-like receptor family pyrin domain-containing 3
GSDMD: Gasdermin D
IL-1β: Interleukin-1 beta
ROS: Reactive oxygen species
HIF-1α: Hypoxia-inducible factor-1 alpha
miRNA: MicroRNA
TNF-α: Tumor necrosis factor-alpha
MDA: Malondialdehyde
HFD: High-fat diet
STZ: Streptozotocin
CMC: Carboxymethyl cellulose
PBS: Phosphate-buffered saline
HOMA-IR: Homeostatic model assessment of insulin resistance
HDL: High-density lipoprotein
LDL: Low-density lipoprotein
LDH: Lactate dehydrogenase
BUN: Blood urea nitrogen
GSH: Reduced glutathione
KW/BW: Kidney weight/body weight
ECM: Extracellular matrix
